# Pharmacokinetic Properties of Three Forms of Vaginal Progesterone Administered in Either Single Or Multiple Dose Regimen in Healthy Post-menopausal Chinese Women

**DOI:** 10.3389/fphar.2017.00212

**Published:** 2017-04-20

**Authors:** Guolan Wu, Junchun Chen, Xingjiang Hu, Huili Zhou, Jian Liu, Duo Lv, Lihua Wu, Jianzhong Shentu

**Affiliations:** ^1^Research Center for Clinical Pharmacy, State Key Laboratory for Diagnosis and Treatment of Infectious Disease, First Affiliated Hospital, College of Medicine, Zhejiang UniversityHangzhou, China; ^2^Department of Education, First Affiliated Hospital, College of Medicine, Zhejiang UniversityHangzhou, China

**Keywords:** pharmacokinetics, vaginal progesterone, safety, bioavailability, healthy post-menopausal women

## Abstract

**Objective:** A generic vaginal progesterone gel has recently been developed in China. Little is known about its pharmacokinetic properties in Chinese subjects. The purpose of our study was to investigate the pharmacokinetics of three forms of vaginal progesterone gel (test formulations at 4 and 8% strength vs. a reference formulation: Crinone 8%) in Chinese healthy post-menopausal women.

**Methods:** This study consisted of two parts study. The part 1 study was a single-center, open-label, 3-period study. Twelve healthy post-menopausal women were to evaluate the safety and pharmacokinetics of 45 mg vaginal progesterone gel (Test 4%) following single dose and multiple doses administered once every other day (q.o.d.) for six times or once daily (q.d.) for 6 days. The part 2 study was a randomized, open-label, 3-stage crossover study. Twelve post-menopausal women received 90 mg vaginal progesterone gel (Test 8%) or 90 mg Crinone (Reference 8%) following single dose and multiple doses (q.o.d. or q.d.). Plasma concentrations of progesterone were measured up to 72 h by using a validated liquid chromatography tandem-mass spectrometry method. The primary pharmacokinetic parameters, maximum plasma concentration (*C*_max_) and area under the plasma concentration–time curve (AUC) from time zero to last measurable concentration (AUC_0-t_) and extrapolated to infinity (AUC_0-∞_) were compared by an analysis of variance using log-transformed data.

**Results:** Totally 24 subjects were enrolled in and completed the study. Following single dose, The geometric mean *C*_max_ values for Test 4%, Test 8%, and Crinone 8% were 6.35, 10.34, 10.45 ng/mL, and their geometric mean AUC_0-t_ (AUC_0-∞_) were 113.73 (118.00), 169.39 (173.98), and 190.07 (201.13) ng⋅h/mL, respectively. The mean *T*_1/2_ values of progesterone were 11.00, 10.92, and 11.40 h, respectively. For 8% test formulation vs. reference, the 90% CIs of the least squares mean test/reference ratios of *C*_max_, AUC_0-t_, and AUC_0-∞_ were 78.32–124.85%, 54.31–146.24%, and 53.64–137.75, respectively. The most frequent adverse events were increased vaginal secretions, most of which were of mild intensity and considered related to treatment.

**Conclusion:** Results with single and multiple doses of vaginal progesterone gel suggest similar pharmacokinetics properties between test formulations and Crinone 8%. Overall, vaginal progesterone gel was well tolerated.

## Introduction

Progesterone is a natural steroid hormone. Currently, it is used to promote fertility and also has been prescribed for induction amenorrhoea, regular bleeding, and subatrophy or full secretory changes of the endometrium, by modifying the dose, duration of treatment and the route of administration (Moyer et al., 1993; de Lignieres et al., 1995; Chakravarty et al., 2005; Silverberg et al., 2012; Sator et al., 2013).

Available progesterone preparations include oral, intramuscular (i.m.), and, more recently, vaginal formulations (Miles et al., 1994; Stanczyk, 1999; Sator et al., 2013). The convenience of oral administration is attractive. However, orally delivered progesterone is rapidly cleared by first-pass hepatic metabolism and this result in poor bioavailability (Nahoul et al., 1993; Simon et al., 1993; Stanczyk, 1999). Consequently, evidence has indicated that orally administered progesterone has lesser efficacy (lower pregnancy rates) when used for luteal support in ART, compared with either the IM or vaginal route (Cometti, 2015). To compensate for this low bioavailability, oral administration requires high doses which associated with systemic adverse effects, including drowsiness, flushing, and nausea (Tavaniotou et al., 2000). Furthermore, metabolites of orally administered progesterone may induce significant hypnotic effects (Miles et al., 1994). The i.m. route reliably achieves serum levels of progesterone encountered in the menstrual cycle luteal phase but can cause patient discomfort, pain, inflammatory reaction at the injection site, sterile abscesses, and possible infection (Stanczyk, 1999; Tavaniotou et al., 2000; Propst et al., 2001; Penzias, 2002; Phy et al., 2003; Pabuccu and Akar, 2005; Zarutskie and Phillips, 2009; Check, 2012). The vaginal route has recently gained attention because it avoids the variable absorption and high first-pass hepatic metabolism after oral ingestion, while also preventing the uncomfortable, often painful, IM injection. Moreover, it is easily administered by the patient.

Crinone 8% (Serono Laboratories, Norwell, MA, USA) is a vaginal gel containing 90 mg progesterone in a bioadhesive polycarbophil base that confers prolonged-release properties. Several studies on its pharmacokinetic properties have been carried out in healthy reproductive aged female subjects (Nahoul et al., 1993; Blake et al., 2010) and post-menopausal women (Levine and Watson, 2000). Following vaginal administration, peak plasma concentration (*C*_max_) were reached approximately 5–7 h. Vaginal application can result in sustained plasma concentrations (Kimzey et al., 1991; Norman et al., 1991; Archer et al., 1995). Although the pharmacokinetic characteristics of the drug have been studied in other populations previously, very little data are available describing these properties in Chinese population. The aim of the present study was to demonstrate the comparative pharmacokinetic parameters of the generic vaginal gel progesterone formulation (at 4 and 8% strength) and a previously marketed vaginal gel progesterone formulation (Crinone 8%) in Chinese healthy post-menopausal women. The study was conducted to meet China Food and Drug Administration requirements for marketing of the new generic formulation. These results will facilitate the understanding of pharmacological characteristics of progesterone and provide a basis for better clinical use of the drug in Chinese population in the future.

## Materials and Methods

This was a single-center, open-label, 2-parts (part 1and 2) trial conducted in healthy post-menopausal women from October 2010 to June 2011. The study protocol was approved by an independent ethics committee, and was conducted in accordance with local regulations and the Declaration of Helsinki and Good Clinical Practice. All subjects provided written informed consent before entering the study.

### Subjects

Eligible women, aged 40–65 years, in natural menopause for at least 1 year, with a body mass index (BMI) of 18–25 kg/m^2^ were included in the study. Each subject underwent a screening examination, including medical history, physical examination, and laboratory tests. Subjects were required to have a plasma progesterone concentration of not greater than 0.1 ng/mL at screening visit. Subjects were excluded from study participation if they had any surgical or medical condition, which, in the investigator’s opinion, might interfere with the absorption, distribution, metabolism, or excretion of study drug. The subjects had not received any medications within 14 days prior to the current study and had not participated in any study within 3 months prior to the current study. Subjects who had a history of hypersensitivity, allergy, and serious adverse drug reaction were excluded from the study. Subjects with positive tests for human immunodeficiency virus, hepatitis B virus surface antigen, or anti-hepatitis C virus antibody were also excluded.

### Study Design and Treatments

**FIGURE 1:**
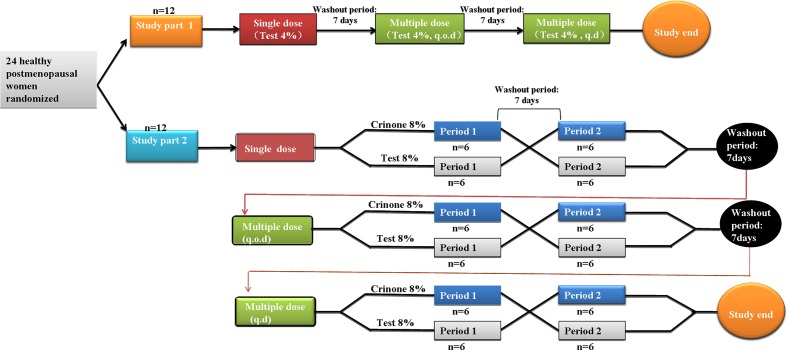
**Study design**.

This was a single-center, randomized, three-arm, pharmacokinetic study. Subjects were randomly assigned using a computer-generated list to participate in one of the three study treatment groups, according to the computer-generated randomization list. Six blocks of four random numbers were generated following the ratio of 2:1:1 for the treatment allocation (Test4%, TR8%, and RT8%, respectively). Each treatment period were separated by a 7-days washout period (at least five times) the mean elimination half-life (∼35 h) after receiving progesterone gel (Prochieve^®^ 8%) (Fleet Laboratories Ltd, 2004). As shown in **Figure 1**, the part 1 study was a single-center, open-label, 3-period study. Twelve healthy post-menopausal women were enrolled to evaluate the safety and pharmacokinetics of 45 mg vaginal progesterone gel (Test 4%) following single dose and multiple doses administered every other day (q.o.d.) for six times or once daily (q.d.) for 6 days. The part 2 study was a randomized, open-label, 3-stage crossover study. Twelve post-menopausal women received 90 mg vaginal progesterone gel (Test 8%) or 90 mg Crinone (Reference 8%) following single dose and multiple doses (every other day dosing or daily dosing). Each subject could only participate in either study part 1 or part 2. All subjects attended the study center on the evening before dosing. A qualified nurse or physician administered progesterone gel to the subject while she was in a reclining position on dosing day at ∼ 7:00 AM. Time of dosing was recorded for each subject. Subjects remained seated or recumbent for 2 h after dosing. The resumption of normal activities, excluding strenuous exercise, was allowed 4 h after dosing. During each period subjects remained in the study center until blood samples had been taken 72 h after last dosing. Carbonated drinks, alcohol, caffeine, tobacco (or nicotine-containing products), or grapefruit (or related citrus fruit) was not permitted during the study.

For both parts of this study, vaginal progesterone gel (test formulations at 4% strength, 45 mg, lot 100701, and test formulations at 8% strength, 90 mg, lot 100501) were supplied by Zhejiang Xianju pharmaceutical Co., Ltd (Xianju, China). Crinone 8% (90 mg, lot C09103/k) were manufactured by Fleet Laboratories Limited, Watford, UK, for Columbia Laboratories, Livingston, NJ, USA.

### Pharmacokinetic Evaluation

For the single-dose phase, blood samples (3 mL) were collected from an indwelling venous catheter into heparinized tubes at -1.0, -0.5, 0, 0.5, 1.0, 1.5, 2.0, 4.0, 6.0, 8.0, 12.0, 24.0, 36.0, 48.0, and 72.0 h. Within 30 min of collection, samples were centrifuged for 15 min at 4000 rpm and at 4°C and stored at -40°C until analysis.

For the multiple-dose phase(4 or 8%, q.o.d or q.d.), blood samples were collected at 0.5, 1.0, 1.5, 2.0, 4.0, 6.0, 8.0, 12.0, 24.0, 36.0, 48.0, and 72.0 h post the last dose. Additional blood samples were obtained before the 4th, 5th, and 6th dose administration to determine the plasma drug concentration at steady state. Other experimental conditions were the same as in single-dose phase.

Plasma concentration of progesterone was analyzed following solid phase extraction (medroxyprogesterone as internal standard) by liquid chromatography tandem-mass spectrometry (LC-MS/MS), which was validated in according to US Food and Drug Administration guidelines on bioanalytical method validation (U.S. Food And Drug Administration, 2001). Briefly, progesterone plasma concentrations were measured by LC-MS/MS using a ZORBAX SB-C18 4.6 × 100 mm, 1.8 μm column. The mobile phase was methanol containing 5 mM ammonium formate – water (90/10, v/v). Calibration curve was constructed at concentrations of 0.0981–39.2 ng/mL. The intra- and inter-day precision (relative standard deviation, %RSD) was ≤4.70% and ≤5.85%, respectively. The intra- and inter-day recovery rates were 96.3–100.4% and 95.18–98.82%, respectively.

### Safety Assessment

Safety evaluations included adverse event counts, monitoring for serious adverse events (SAEs) with the severity and relationship to the study drug and regular monitoring of vital signs, physical condition, clinical laboratory tests (e.g., routine hematology, blood biochemistry and urine analysis) and 12-lead electrocardiogram. The physicians were responsible for determining the clinical significance of the adverse events (AEs). All information, including undesirable symptoms or medical conditions after dosing, were recorded on the case-report form by investigators regardless of the suspected relationship to the study drugs.

### Pharmacokinetic Analysis

The population for pharmacokinetic analysis consisted of all subjects who had received at least one dose of study medication and had an evaluable pharmacokinetic profile. Pharmacokinetic parameters were calculated by non-compartment method using WinNonlin 6.3 software (Pharsight Corporation; Sunnyvale, CA, USA). The primary outcome measure was the evaluation of the bioavailability of the test products and the reference product, assessed as extent of exposure (AUC, area under the curve) after single administration (AUC_0-t_) and multiple administrations (AUC_ss_) of the three formulations. The secondary outcomes were the pharmacokinetic parameters: maximum serum concentration (*C*_max_), area under the time-concentration curve from first time point extrapolated to infinity (AUC_0-∞_), time to maximum plasma concentration (*T*_max_) and *t*_1/2_ after a single dose, and maximum and minimum plasma concentration after last dose (*C*_ss,max_ and *C*_ss,min_) and plasma peak concentration time in the dosing interval after last dose (*T*_ss,max_) after multiple doses.

### Statistical Analysis

Sample sizes for each individual study were chosen to ensure that there were sufficient PK data, and were based on previous experience with other compounds, not on formal statistical considerations. Data points below the lower limit of quantification (LOQ) were substituted by zero for the calculation of mean values. Pharmacokinetic parameters were expressed using arithmetic means and standard deviations, except for *T*_max_, for which median values and ranges are reported. To investigate dose dependent changes in pharmacokinetics, analysis of variance (ANOVA) was used to compare pharmacokinetic parameters (*C*_max_/dose, AUC/dose, and *t*_1/2_) at different dose levels for three different dosing regimens. *t*-test was used to determine whether the multiple-dose pharmacokinetic parameters were consistent with those in the single-dose phase. The results for *T*_max_ were evaluated using Wilcoxon rank sum analysis. *P* < 0.05 was considered statistically significant.

To assess the relative bioavailability of 90 mg vaginal progesterone gel (Test 8%) or 90 mg Crinone (Reference 8%), values for AUC_0-t_ (AUC_0-t,ss_) and *C*_max_ (*C*_max,ss_) were log transformed (natural logarithm) prior to fitting an ANOVA model that included “sequence,” “period” and “treatment” as fixed effects and “subjects within sequence” as a random effect. The difference between the expected means for log (T) – log (R) was estimated by the difference in the corresponding least square means (point estimate), and two sided 90% CIs based on the t-distribution were computed. These values were back transformed to the original scale to give the point estimator (geometric mean; gMean) and 2-sided 90% CIs for the intra-subject GMR between response under test and reference conditions.

## Results

### Study Participants

**FIGURE 2:**
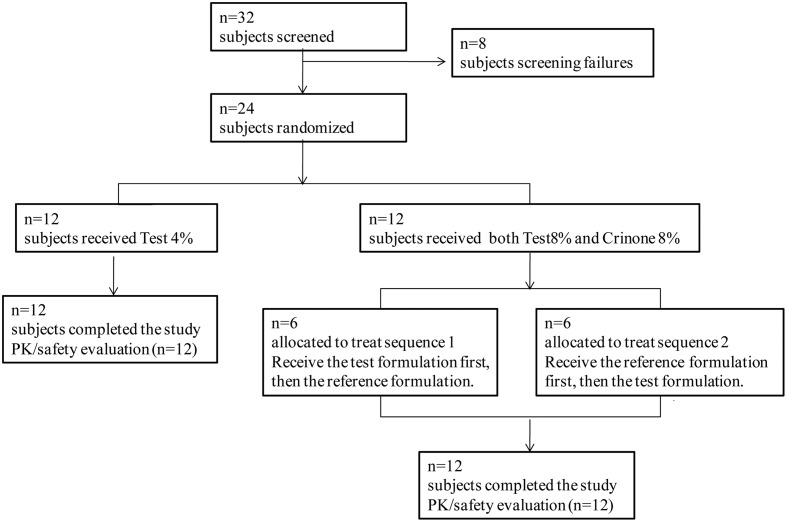
**Diagram of the disposition of subjects**.

Thirty-two post-menopausal women were assessed for study eligibility and 24 of them were randomized to the study and received the allocated treatment according to the protocol (**Figure 2**). The eight subjects not randomized were screening failures. In study part 1, 12 healthy post-menopausal women were enrolled in and completed the study. The mean age (range) of subjects was 55.4(49–61) years, mean body weight (range) was 54.8(49.0–65.0) kg, mean height (range) was 1.59(1.53–1.67) m and mean BMI (range) was 20.6(19.3–23.8) kg/m^2^. In study part 2, 12 healthy post-menopausal women were 51–63 years old and had a mean body weight of 57.2 ± 3.1 kg and a mean BMI of 22.8 ± 0.8 kg/m^2^. Demographic characteristics for the subjects are summarized in **Table 1**.

**Table 1:** Baseline characteristics of the randomized study population (*n* = 24).

Characteristic	Study part 1	Study part 2
	Female (*n* = 12)	Female (*n* = 12)
Age, mean (range), years	55.4 (49-61)	56.6 (51-63)
Height, mean (range), m^2^	1.59 (1.53-1.67)	1.58 (1.52-1.65)
Weight, mean (range), kg	54.8 (47.0-65.0)	57.2 (52.0-62.0)
BMI, mean (range), kg/m^2^	21.6 (19.3-23.8)	22.8 (21.4-23.8)
Menopausal years, mean (*SD*), years	6.0 (3.02)	6.6 (4.29)

### Safety Results

In total, nine subjects (37.5%) reported ≥1 AE: six subjects in part 1 and three subjects in part 2. All of the AEs were considered by the investigator to have been of mild intensity and occurred during the multiple-dose period. A summary of the numbers of treatment-emergent adverse events (TEAEs) during study period is given in **Table 2**. No SAEs occurred during the study, and all subjects were in good compliance with the protocol. No clinically significant laboratory results or trends were reported for any dose at any time point. No clinically meaningful changes in physical examinations, vital signs, or ECG studies were apparent during the study.

**Table 2:** Summary of adverse events (AEs) during the study period (*n* = 24).

Adverse events	Study part 1 (*n* = 12)		Study part 2 (*n* = 12)	
	All AEs	Drug-related AEs	All AEs	Drug-related AEs
Total	6 (6)	6 (6)	3 (4)	3 (4)
Lower abdominal pain	1 (1)	1 (1)	2 (2)	2 (2)
Abdominal distension	0 (0)	0 (00)	1 (1)	1 (1)
Lumbar acid	1 (1)	1 (1)	0 (0)	0 (0)
Increased vaginal secretions	2 (2)	2 (2)	0 (0)	0 (0)
Colporrhagia	1 (1)	1 (1)	0 (0)	0 (0)
Urinary tract infection	1 (1)	1 (1)	0 (0)	0 (0)

*Data presented as number of subjects (number of events)*.

### Pharmacokinetic Profiles

#### Single-dose Administration in Healthy Volunteers (45–90 mg, Delivered Vaginally)

**FIGURE 3:**
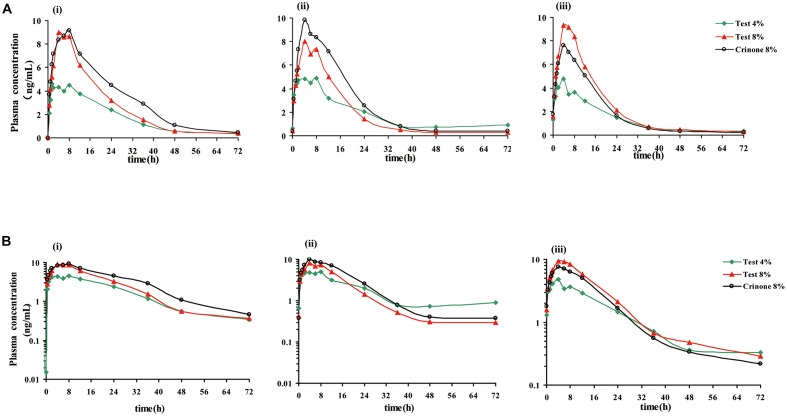
**Mean plasma progesterone concentration vs. time curves after receiving three forms of vaginal progesterone gel**. Mean plasma progesterone level after single dose (i), mean plasma progesterone level following the last administration (every other day dosing, ii), mean plasma progesterone level following the last administration (daily dosing, iii). Linear scale **(A)**, semi-log scale **(B)**.

The mean plasma concentration-time profile of progesterone following single-dose vaginal administration of all three formulations is shown in **Figure 3**. Pharmacokinetic parameters are presented in **Table 3**. Variability between subjects was large in three formulations and geometric CV was typically greater than 50%.

**Table 3:** Pharmacokinetic parameters of progesterone after single dose of vaginal progesterone gel.

Parameter (unit)	Single dose^a^		
	45 mg test	90 mg test	90 mg Crinone
	(*n* = 12)	(*n* = 12)	(*n* = 12)
AUC_0-24h_ (ng⋅h/mL)	82.06 (26.25)	132.90 (45.06)	140.28 (67.10)
AUC_0-72h_ (ng⋅h/mL)	113.73 (47.2)	169.39 (58.8)	190.07 (75.3)
AUC_0-∞_ (ng⋅h/mL)	118.01 (52.1)	173.98 (60.3)	201.13 (71.6)
*C*_max_ (ng/mL)	6.35 (25.5)	10.34 (30.0)	10.45 (36.4)
*T*_max_ (h)^b^	6.6 (1.5-24.0)	4.0 (2.0-12.0)	6.2 (1.0-24.0)
*t*_1/2_ (h)	10.13 (47.4)	9.42 (85.6)	10.1 (66.3)

^a^*Results are presented as geometric mean (CV%) unless stated*.

^b^*These values are expressed as median(range)*.

*AUC_0-*t*_, area under the plasma concentration-time curve from zero time to time of the last quantifiable drug plasma concentration; AUC_0-∞_, AUC from time zero extrapolated to infinity; *C*_*max*_, maximum plasma concentration; *T*_*max*_, time to maximum plasma concentration; *t*_*1*/2_, elimination half-life*.

Progesterone exposure (AUC_0-∞_ and *C*_max_) increased with dose, while the *T*_max_ and *t*_1/2_ were comparable between the 45 mg and 90 mg doses of progesterone. For the relationship between non-compartmental parameters (AUC_0-∞_ and/or AUC_0-72h_ and *C*_max_) and dose after administration of the test formulations (45 and 90 mg), analysis results indicated that the 90% CIs of the ratios (90 mg test/45 mg test) of the geometric mean were 1.173–2.252, 1.227–2.140 and 1.432–2.043 for AUC_0-∞_, AUC_0-72h_ and *C*_max_, respectively. This indicated lesser-than-proportional exposure with dose.

Progesterone bioavailability after single dose administration of the test (90 mg test) and reference formulation (90 mg Crinone) was similar. The geometric mean ratios (T/R) and 90% confidence intervals for *C*_max_, AUC_0-t_ and AUC_0-∞_ are summarized in **Table 4**. No significant differences in *t*_1/2_, *T*_max_, *C*_max_/dose, or AUC/dose were found among the three formulations.

**Table 4:** Geometric mean ratios (GMR) T/R, 90% confidence intervals (90% CI) for *C*_max_, AUCs following single dose or multiple doses (q.o.d or q.d. dosing regiment) of Test (90 mg test) and reference formulation (90 mg Crinone) to12 healthy post-menopausal women.

Administration	Parameters	Geometric	90%
		mean ratio	geometric
		(%)*^a^*	CI (%)
Single dose	AUC_0-72h_ (ng⋅h/mL)	89.12	54.31-146.24
	AUC_0-∞_ (ng⋅h/mL)	85.96	53.64-137.75
	*C*_max_ (ng/mL)	98.88	78.32-124.85
Multiple dose (q.o.d)	AUC_0-τ,ss_ (ng⋅h/mL)	76.43	61.35-95.22
	AUC_0-72h_ (ng⋅h/mL)	75.09	60.26-93.57
	*C*_max,ss_ (ng/mL)	84.79	68.21-105.40
Multiple dose (q.d.)	AUC_0-τ,ss_ (ng⋅h/mL)	123.35	101.78-149.49
	AUC_0-72h_ (ng⋅h/mL)	124.28	98.98-156.06
	*C*_max,ss_ (ng/mL)	118.48	97.70-143.68

^a^*Log-transformed data, ratio of least-square geometric means (test/reference) based on ANOVA model. *C*_*max*_, maximum plasma concentration; *C*_*max,ss*_, maximum plasma concentration at the steady state; AUC_0-*t*_, area under the plasma concentration-time curve from zero time to time of the last quantifiable drug plasma concentration; AUC_0-τ*,ss*_; area under concentration–time curve over a uniform dosing interval τ.*

#### Multiple-dose Administration in Healthy Volunteers (45–90 mg, Delivered Vaginally, q.o.d or q.d. for six doses)

The mean plasma concentration-time profiles of progesterone in different treatment groups are presented in **Figure 3**, and the pharmacokinetic parameters are summarized in **Table 5**. Steady state, based on plasma concentration of drug in pre-4th, 5th, and 6th dose samples, was achieved after administration of progesterone formulations for six consecutive doses. Following the last administration(q.o.d dosing), *C*_max,ss_ were reached 4.62 ± 2.90, 6.12 ± 3.30, and 5.33 ± 1.97 ng/mL after administration of Test 4%, Test 8%, and Crinone 8%, respectively. Comparing with q.o.d. dosing regiment, the values for *T*_max,ss_ of each formulation in the q.d. dosing regiment were very similar. Afterward, plasma concentrations declined in a biphasic manner, with a rapid distribution phase and slower elimination phase (**Figure 3**). As shown in **Table 5**, plasma *t*_1/2_ was independent of the dose and dosing duration, averaging 10.98 h (range, 3.9∼34.9 h). In the daily dosing regiment study, the mean *C*_max,ss_, AUC_τ,ss_ of 90 mg test formulation were 1.85- and 1.79-fold higher than the corresponding *C*_max,ss_, AUC_τ,ss_ of 45 mg test formulation, respectively. However, this exposure at steady state (*C*_max,ss_, AUC_τ,ss_) to dose increase ratios were much lower in the every other day dosing regiment: *C*_max,ss_ increased only by 1.40-fold and AUC_τ,ss_ increased only by 1.13-fold. The accumulation ratio was to found to be in the range of 1.01 and 1.18, indicating that there was no accumulation after multiple doses of vaginal progesterone gel.

**Table 5:** Pharmacokinetic parameters of progesterone at the steady-state after administration three formulations of vaginal progesterone gel.

Parameters	Multiple doses^a^					
	Every other day dosing			Daily dosing		
	45 mg test	90 mg test	90 mg Crinone	45 mg test	90 mg test	90 mg Crinone
	(*n* = 12)	(*n* = 12)	(*n* = 12)	(*n* = 12)	(*n* = 12)	(*n* = 12)
AUC_0-τ,ss_	98.19 (43.2)	127.05 (24)	166.52 (40.8)	64.39 (49.2)	133.87 (20.4)	108.53 (31.2)
*C*_max,ss_(ng/mL)	6.22 (40.6)	8.95 (26)	10.56 (35.4)	5.07 (61.7)	10.12 (27.4)	8.54 (26.6)
*T*_max,ss_ (h)*^b^*	4.0 (0.5-8)	5.0 (1.5-12)	5.0 (2-8)	3.0 (0.5-24)	6.0 (4-12)	5.0 (1.5-12)
*C*_min,ss_(ng/ mL)	0.55 (146.4)	0.31 (156.3)	0.31 (102.9)	0.94 (120.9)	1.26 (91)	1.45 (77.6)
*C*_avg_(ng/ mL)	2.05 (43.1)	2.65 (24.0)	3.47 (40.8)	2.68 (49.2)	5.58 (20.4)	4.52 (31.2)
*t*_1/2_(h)	10.92 (121.3)	7.62 (53)	11.08 (48.9)	10.64 (58.5)	9.9 (89.5)	10.13 (71.9)
AUC_0-72h__(_ng⋅h/mL)	105.15 (51.7)	129.76 (25.3)	172.87 (39.8)	81.02 (67.2)	159.06 (26.2)	127.98 (36.7)
AUC_0-∞_(ng⋅h/mL)	115.34 (64)	132.24 (25.9)	177.59 (38.9)	85.27 (65.1)	164.13 (29.7)	130.53 (37.3)
DF	2.51 (39.3)	3.14 (35.4)	2.9 (34.1)	1.28 (61.5)	1.51 (31.5)	1.5 (33.6)

^a^*Results are presented as geometric mean(CV%) unless stated*.

^b^*These values are expressed as median(range)*.

*C_*max,ss*_, maximum plasma concentration at the steady state; *T*_*max,ss*_, time to maximum plasma concentration at the steady state; *C*_*min,ss*_, minimum plasma concentration at the steady state; AUC_0-τ*,ss*_, area under concentration–time curve over a uniform dosing interval τ; AUC_0-*t*_, area under the plasma concentration-time curve from zero time to time of the last quantifiable drug plasma concentration; AUC_0-∞_, AUC from time zero extrapolated to infinity; *t*_1/2_, elimination half-life; DF, degree of fluctuation*.

## Discussion

To our knowledge, this is the first published study to characterize the tolerability and pharmacokinetic profile of progesterone after vaginal administration of single and multiple doses in Chinese healthy post-menopausal women. Also, our study shows a direct comparison of the pharmacokinetics of progesterone in three different formulations of progesterone gel. In addition, three dosing regiments (single dosing, daily dosing and every other day dosing) were tested. In the current study, the three forms of vaginal progesterone gel were generally safe and well tolerated at the dose regimens studied.

Previous studies reported *C*_max_ value of 10.51 ng/mL following vaginal administration of Crinone 8% to healthy post-menopausal women (Levine and Watson, 2000). This value is comparable with those obtained in the present study, the mean *C*_max_ values for Crinone 8%, Test 8%, and Test 4% were 11.13, 10.73, and 6.52 ng/mL, respectively. *T*_max_ in our study [mean (SD), 7.37 (6.16) h for Crinone 8%, 5.33 (3.11) h for Test 8% and 6.50 (6.64) h for Test 4%] was also similar to that obtained in previous study, which described *T*_max_ value of 7.67 (3.67) h (Levine and Watson, 2000). However, AUC_0-24_ of three different formulations [mean (SD), 161.76 (77.04) ng⋅h/mL for Crinone 8%, 143.45 (53.01) ng⋅h/mL for Test 8% and 84.65 (22.59) ng⋅h/mL for Test 4%] were higher than the value reported in the literature [Crinone 8% 133.26 (14.61) ng⋅h/mL]. The *t*_1/2z_ was not affected by formulation type or repeat dosing, and *C*_max_ and AUC_0-24_ values under steady-state conditions were similar to those obtained after single administration of the same doses, thus no accumulation of progesterone took place.

Even though demonstrating bioequivalence of the formulations is not the purpose of present study, the results may help to design a pivotal study appropriately. In present study, inter-individual variability in *C*_max_ and AUC values was considerable, with CV% ranging from 20.4 to 67.10% for each treatment group. The CV_intra-subject_, for *C*_max_ and AUC_S_, could obtain from ANOVA analysis using the following equation: CV_intra-subject_ = SQRT (EXP (s^2^)-1) (Ramirez et al., 2013). The CV_intra-subject_ of *C*_max_ and AUC were estimated to be 21 and 44%, respectively. Thus, applicants may consider using a reference-scaled average BE approach for progesterone.

In the present study, a specific method LC-MS/MS was used to measure the plasma concentration of progesterone after vaginal administration. Further, additional blood samples were obtained before dosing to determine the plasma progesterone concentration in the healthy post-menopausal women. Then, only subjects with the plasma progesterone level below the LOQ could be included in the study. Taken together, the present study was able to provide valuable information on the plasma pharmacokinetics of progesterone after the single and repeated administration of progesterone gel by two different dosing regimens.

However, the study was conducted in healthy volunteers with very similar BMIs. Caution should be exercised when extrapolating these data to patients and special groups, especially patients with renal disease or infertile women. The sample size of the present study was also relatively small, which could have led to possible bias and insufficient accuracy.

## Conclusion

In this study, pharmacokinetic profiles of progesterone after single doses of Test 4%, Test 8% and Crinone 8% were determined. Steady state was achieved after six repeated dose administration of vaginal progesterone gel. No evidence of drug accumulation in plasma was seen. All dose regimens were well tolerated in healthy post-menopausal women, with only a small number of mild AEs.

## Ethics Statement

This study was carried out in accordance with the recommendations of the Guideline for Good Clinical Practice and the Guideline for Pharmacokinetics studies. All subjects gave written informed consent in accordance with the Declaration of Helsinki. The protocol was approved by the ethics committee of the First Affiliated Hospital, College of Medicine, Zhejiang University (approval No.:2010-EC-13).

## Author Contributions

GW and JC was responsible for the study design, data acquisition, and data analysis, as well as writing and revising the article. JS approved the final version and agreed to be accountable for all aspects of the work. HZ and XH took responsibility for bioanalytical components of the study. LW, JL, and DL contributed to the pharmacokinetics analysis, the statistical analysis, and data interpretation. All authors listed were responsible for critically revising the manuscript for important intellectual content and approval of the final version to be published.

## Conflict of Interest Statement

The authors declare that the research was conducted in the absence of any commercial or financial relationships that could be construed as a potential conflict of interest.
